# Sarcoidosis activates diverse transcriptional programs in bronchoalveolar lavage cells

**DOI:** 10.1186/s12931-016-0411-y

**Published:** 2016-07-26

**Authors:** Sina A. Gharib, Anagha Malur, Isham Huizar, Barbara P. Barna, Mani S. Kavuru, Lynn M. Schnapp, Mary Jane Thomassen

**Affiliations:** 1 Division of Pulmonary and Critical Care Medicine, Department of Medicine, University of Washington, Seattle, WA USA; 2 Computational Medicine Core, Center for Lung Biology, Department of Medicine, University of Washington, Seattle, WA USA; 3 Division of Pulmonary, Critical Care and Sleep Medicine, Brody School of Medicine, East Carolina University, Greenville, NC USA; 4 Division of Pulmonary and Critical Care Medicine, Texas Technical University Health Science Center, Lubbock, TX USA; 5 Division of Pulmonary and Critical Care Medicine, Thomas Jefferson University Hospital, Philadelphia, PA USA; 6 Pulmonary, Critical Care, Allergy and Sleep Medicine, Medical University of South Carolina, Charleston, SC USA; 7 850 Republican, Box 358052, Seattle, WA 98109-4725 USA

**Keywords:** Sarcoidosis, Microarray, Proteasome, Network analysis

## Abstract

**Background:**

Sarcoidosis is a multisystem immuno-inflammatory disorder of unknown etiology that most commonly involves the lungs. We hypothesized that an unbiased approach to identify pathways activated in bronchoalveolar lavage (BAL) cells can shed light on the pathogenesis of this complex disease.

**Methods:**

We recruited 15 patients with various stages of sarcoidosis and 12 healthy controls. All subjects underwent bronchoscopy with lavage. For each subject, total RNA was extracted from BAL cells and hybridized to an Affymetrix U133A microarray. Rigorous statistical methods were applied to identify differential gene expression between subjects with sarcoidosis vs. controls. To better elucidate pathways differentially activated between these groups, we integrated network and gene set enrichment analyses of BAL cell transcriptional profiles.

**Results:**

Sarcoidosis patients were either non-smokers or former smokers, all had lung involvement and only two were on systemic prednisone. Healthy controls were all non-smokers. Comparison of BAL cell gene expression between sarcoidosis and healthy subjects revealed over 1500 differentially expressed genes. Several previously described immune mediators, such as interferon gamma, were upregulated in the sarcoidosis subjects. Using an integrative computational approach we constructed a modular network of over 80 gene sets that were highly enriched in patients with sarcoidosis. Many of these pathways mapped to inflammatory and immune-related processes including adaptive immunity, T-cell signaling, graft vs. host disease, interleukin 12, 23 and 17 signaling. Additionally, we uncovered a close association between the proteasome machinery and adaptive immunity, highlighting a potentially important and targetable relationship in the pathobiology of sarcoidosis.

**Conclusions:**

BAL cells in sarcoidosis are characterized by enrichment of distinct transcriptional programs involved in immunity and proteasomal processes. Our findings add to the growing evidence implicating alveolar resident immune effector cells in the pathogenesis of sarcoidosis and identify specific pathways whose activation may modulate disease progression.

**Electronic supplementary material:**

The online version of this article (doi:10.1186/s12931-016-0411-y) contains supplementary material, which is available to authorized users.

## Background

Sarcoidosis is a heterogeneous disease characterized by noncaseating granulomatous inflammation affecting multiple organs. The lung is the most common site involved in this systemic disease with over 90 % of patients developing pulmonary sarcoidosis [1]. Although the majority of patients have a self-limited course, a significant subset progresses to chronic lung impairment including development of pulmonary fibrosis and hypertension. The etiology of sarcoidosis is unknown and its pathobiology is incompletely understood, making treatment and management challenging [2]. However, a substantial body of evidence implicates dysregulated adaptive immune response against an unknown inciting agent as the hallmark event in the immunopathogenesis of sarcoidosis [2, 3]. In particular, aberrant interactions between alveolar macrophages and T-helper 1 (Th1) cells in conjunction with increased Th1 and other cytokine levels in the airspaces are key features of pulmonary sarcoidosis [3].

To our knowledge, the transcriptional state of immune effecter cells residing within lung airspaces has not been systematically surveyed in sarcoidosis. We hypothesized that an unbiased approach to identify pathways differentially activated in bronchoalveolar lavage (BAL) cells of sarcoidosis patients could shed light on the pathogenesis of this complex disease and reveal previously unrecognized targets for future therapeutic intervention.

## Methods

### Study subjects

Sarcoidosis subjects (*N* = 25) were recruited from patients undergoing routine clinical evaluation for diagnosis of sarcoidosis without acute pulmonary symptoms (Table 1). None had Löfgren’s syndrome [4]. All diagnoses were confirmed by pulmonary histology demonstrating non-necrotizing granulomas in the absence of infection or other etiologies. A healthy group (*N* = 12) composed of individuals with no history of lung disease and no medication usage at time of bronchoscopy was recruited as controls. These healthy individuals volunteered to undergo bronchoscopy as part of an Institutional Review Board-approved research program. The entire protocol was approved by the East Carolina University & Medical Center Institutional Review Board (#05-0581) and written informed consent was obtained from all participants.

**Table 1:** Subject demographics and BAL cell counts

Subject	Gender	Age	Race	Smoking	Stage	Mac (%)	Lymph (%)	Pmn (%)	Eos (%)
Sarc 1	M	52	AA	Former	3	68	32	0	0
Sarc 2	F	32	AA	Former	3	67	30	3	0
Sarc 3	F	39	AA	Never	2	89	10	1	0
Sarc 4	F	61	AA	Never	NA	64	35	1	0
Sarc 5	M	57	AA	Former	3	95	4	0	1
Sarc 6	M	68	AA	Never	4	86	14	0	0
Sarc 7	F	62	AA	Never	0	53	47	0	0
Sarc 8	M	34	AA	Never	2	91	8	1	0
Sarc 9	F	47	AA	Former	4	97	3	0	0
Sarc 10	M	48	AA	Never	2	61	37	2	0
Sarc 11	F	53	C	Never	2	96	4	0	0
Sarc 12	F	39	AA	Never	2	70	30	0	0
Sarc 13	F	42	AA	Former	1	85	15	0	0
Sarc 14	F	46	AA	Never	2	67	9	24	0
Sarc 15	M	49	AA	Former	2	94	5	0	1
Cont 1	M	27	AA	Never	—	92	5	3	0
Cont 2	M	28	AA	Never	—	98	2	0	0
Cont 3	F	51	C	Never	—	92	8	0	0
Cont 4	F	31	AA	Never	—	92	8	0	0
Cont 5	F	26	AA	Never	—	91	4	3	2
Cont 6	F	24	AA	Never	—	95	5	0	0
Cont 7	F	28	AA	Never	—	98	1	0	1
Cont 8	M	28	C	Never	—	98	2	0	0
Cont 9	F	26	AA	Never	—	98	2	0	0
Cont 10	F	27	AA	Never	—	97	3	0	0
Cont 11	M	30	AA	Never	—	95	5	0	0
Cont 12	F	35	AA	Never	—	93	7	0	0

*Sarc* sarcoidosis patient, *Cont* control subject, *AA* African American, *C* Caucasian, *Mac* macrophage, *Lymph* lymphocyte, *Pmn* polymorphonuclear cell, *Eos* eosinophil

### Isolation of BAL cells

Briefly, three separate 50 mL aliquots of 0.89 % sterile saline were instilled into the right middle lobe and lingula for a total volume of 300 mL. Immediately on collection, BAL fluid was centrifuged at 500 g for 10 min at 4 °C. The cell free supernatant was aliquoted and immediately frozen at −70 °C. BAL cells were washed with hanks balanced salt solution, resuspended and counted with a hemocytometer. Mean viability of lavage cells was > 95 % as determined by trypan blue dye exclusion. Cytospin preparations were stained with a modified wright stain for determination of differential cell counts.

### Microarray experiments

Transcriptional profiling was performed on a subset of the study cohort: 15 subjects with sarcoidosis and the 12 controls. For each subject, total RNA from BAL cells was extracted using the RNeasy mini kit (Qiagen, Valencia, CA) and its integrity verified by the Agilent 2100 Bioanalyzer (Agilent Technologies, Santa Clara, CA). Amplified biotin-labeled cRNA was generated from 2 mg of total RNA and hybridized to Affymetrix Human GeneChip U133A 2.0 microarrays (Affymetrix, Santa Clara, CA) and processed according to the manufacturer’s instructions. Background adjustment and quantile normalization of log-transformed intensities was performed using RMA software [5]. Detailed microarray experiment description, meeting Minimum Information About a Microarray Experiment (MIAME) requirements, has been deposited at Gene Expression Omnibus (http://www.ncbi.nlm.nih.gov/geo, GSE75023).

### Microarray data analysis

Initially, we used a form of multidimensional scaling known as correspondence analysis [6] to determine whether global variation in gene expression in BAL cells between sarcoidosis and normal subjects would effectively segregate these two groups. Next, differential gene expression between airspace cells harvested from sarcoidosis vs. normal controls was determined using a Bayesian implementation of the parametric *t*-test [7] coupled with false discovery (FDR) analysis [8]. An FDR < 1 % was chosen to identify significant differential gene expression. We performed two dimensional hierarchical cluster analysis based on Pearson’s correlation to identify association patterns within differentially expressed genes and samples [9].

We applied two levels of functional enrichment analysis to the expression dataset. First we focused on the subset of differentially expressed genes (FDR < 1 %) and used Webgestalt software [10] to identify over-represented Gene Ontology categories [11]. Enrichment *P*-values from this analysis were corrected for multiple testing using an FDR threshold < 1 %. We then undertook a more unbiased and comprehensive approach by applying Gene Set Enrichment Analysis (GSEA) to the entire microarray dataset using over 1300 canonical pathways derived from multiple resources including KEGG, Reactome, Pathway Interaction Database, and Biocarta among others [12]. These pathways have been previously curated by experts and were available through GSEA’s molecular signature database. An FDR < 1 % was used to designate significant enrichment for gene sets. We used Enrichment Map [13], an application within the Cytoscape (v3.1.1) environment, to develop a network-based visualization of the GSEA results [14]. To display connectivity among members of specific enriched gene sets, we performed network analysis using gene product interaction resources (Ingenuity, STRING) and limited the relationships to experimentally verified direct interactions [15, 16]. A schematic overview of our approach is provided in Additional file 1: Figure S1.

### qPCR validation

We confirmed differential expression of selected genes by qPCR using the ABI Prism 7300 Detection System (TaqMan, Applied Biosystems, Foster City, CA). RNA from BAL cell samples were analyzed in duplicate using primer sets for proteasome subunit beta type 8 (PSMB8) (Cat# PPH13734C, Qiagen, Valencia, CA), proteasome subunit beta type 9 (PSMB9) (Cat# PPH13734C, Qiagen) and interferon gamma (IFNG) (Cat# PPH00380C, Qiagen). Threshold cycle (C_T_) values for genes of interest were normalized to a housekeeping gene, glyceraldehyde 3-phosphate dehydrogenase (GAPDH), as previously described [17] and relative expression was calculated using the delta-delta method [18]. Data are presented as fold change in mRNA expression between sarcoidosis patients versus healthy controls. *P*-values were calculated using two-tailed Student’s *t*-test of log_2_-transformed data (Prism v6, GraphPad Software, La Jolla, CA).

### BAL cytokine assays

We evaluated the protein expression of two candidate genes identified from the microarray experiments, IL6 and CCL5 (RANTES), in BAL fluid of sarcoidosis and control subjects using ultrasensitive kits (MesoScale Discovery, Gaithersburg, MD) as per manufacturer’s instructions. The plates were read using the MSD Discovery workbench analyzer and software package. Two groups of sarcoidosis patients were studied: 1) subjects who also underwent microarray analysis of their BAL cells (sarcoidosis 1, *N* = 15); 2) a validation group of patients that were not part of the original microarray study (sarcoidosis 2, *N* = 10). *P*-values were calculated using the nonparametric, two-tailed Mann–Whitney test because the cytokine data were not normally distributed based on Shapiro-Wilk’s normality test (Prism v6, GraphPad Software, La Jolla, CA).

## Results

### Subject characteristics

Microarray analyses were conducted on BAL cells from 15 patients with pulmonary sarcoidosis and compared with 12 healthy controls. Demographic data for each subject is shown in Table 1. Both groups were predominantly African-American and female, and none were active smokers. None of the healthy controls were on medication and only two of the sarcoidosis patients were on systemic steroids. All sarcoidosis subjects had lung involvement with a majority having Scadding chest radiograph stages 2 or 3. BAL cell differential counts in both groups were predominantly macrophages, although several sarcoidosis patients were characterized by significant lymphocytosis. Detailed demographic information is provided in Additional file 2: Table S1.

### Sarcoidosis is associated with distinct transcriptional profiles in BAL cells

**Fig. 1:**
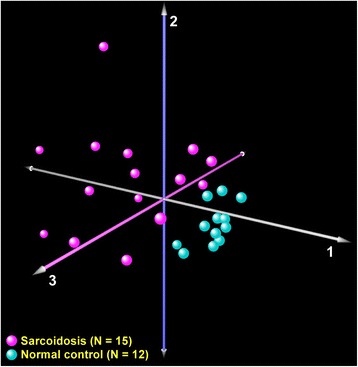
Correspondence analysis of transcriptional profiles between sarcoidosis patients (*magenta*) and normal controls (*cyan*). Each axis captures a proportion of the observed whole-genome expression variability across all subjects. Note that this unbiased analysis segregated the cohort into two groups based on disease status, implying that sarcoidosis induces global changes in BAL cell transcriptome

**Fig. 2:**
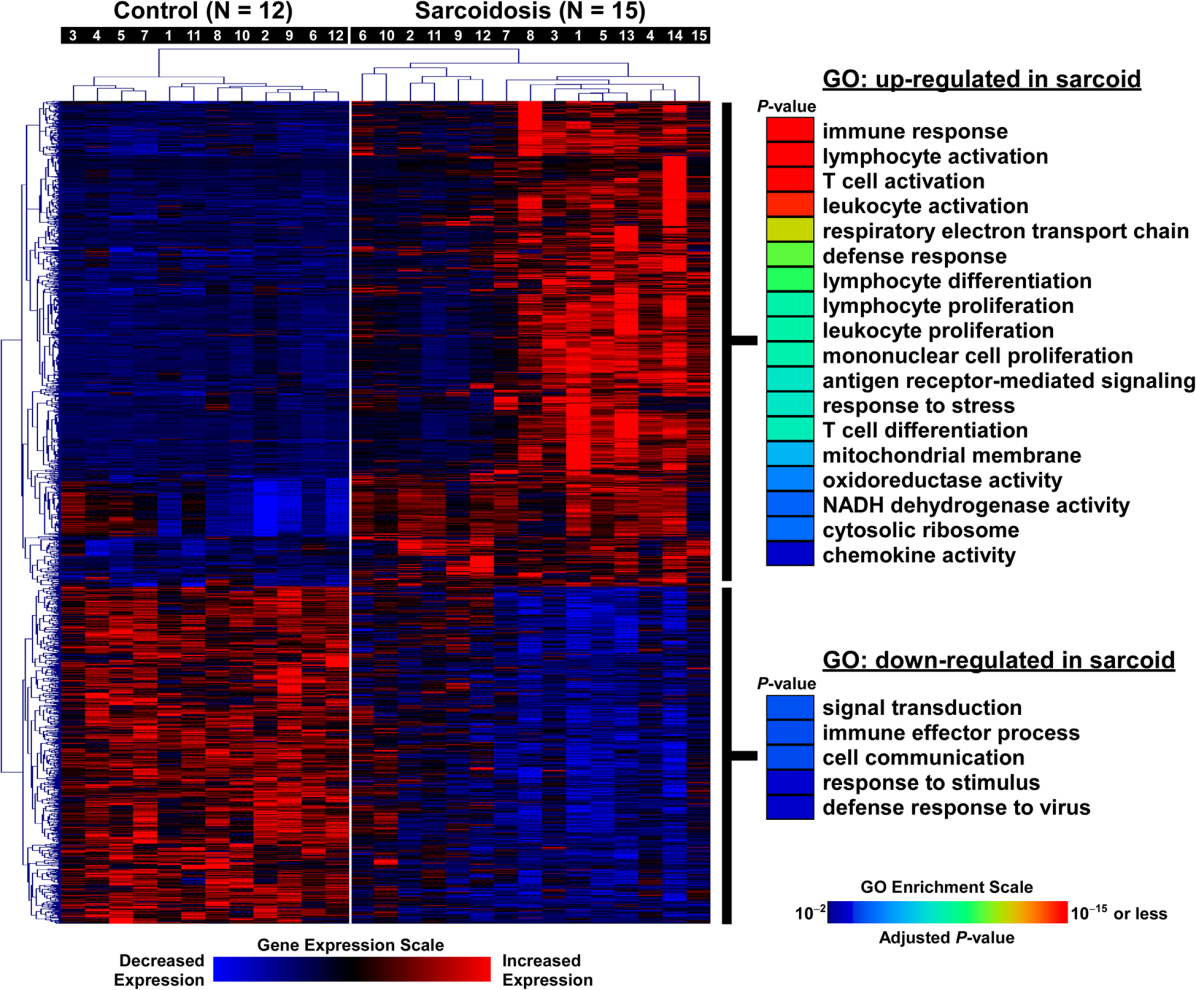
Cluster analysis of differentially expressed genes in BAL cells between sarcoidosis patients and normal controls. Subject numbers correspond to Table 1 descriptions. Significantly over-represented processes among differentially upregulated and downregulated genes in sarcoidosis were identified using Gene Ontology (GO) annotation and are shown on the right panel

Correspondence analysis of the entire microarray dataset revealed that global variation in gene expression across samples segregated sarcoidosis subjects from healthy controls (Fig. 1). This finding implies that the transcriptome of airspace cells is significantly altered in sarcoidosis. Using strict statistical criteria (FDR < 1 %), we identified over 1500 differentially expressed transcripts between the two groups (Fig. 2). Functional enrichment of these genes revealed profound upregulation of many processes involved in immunity and inflammation.

### Sarcoidosis is characterized by activation of widespread transcriptional programs

**Fig. 3:**
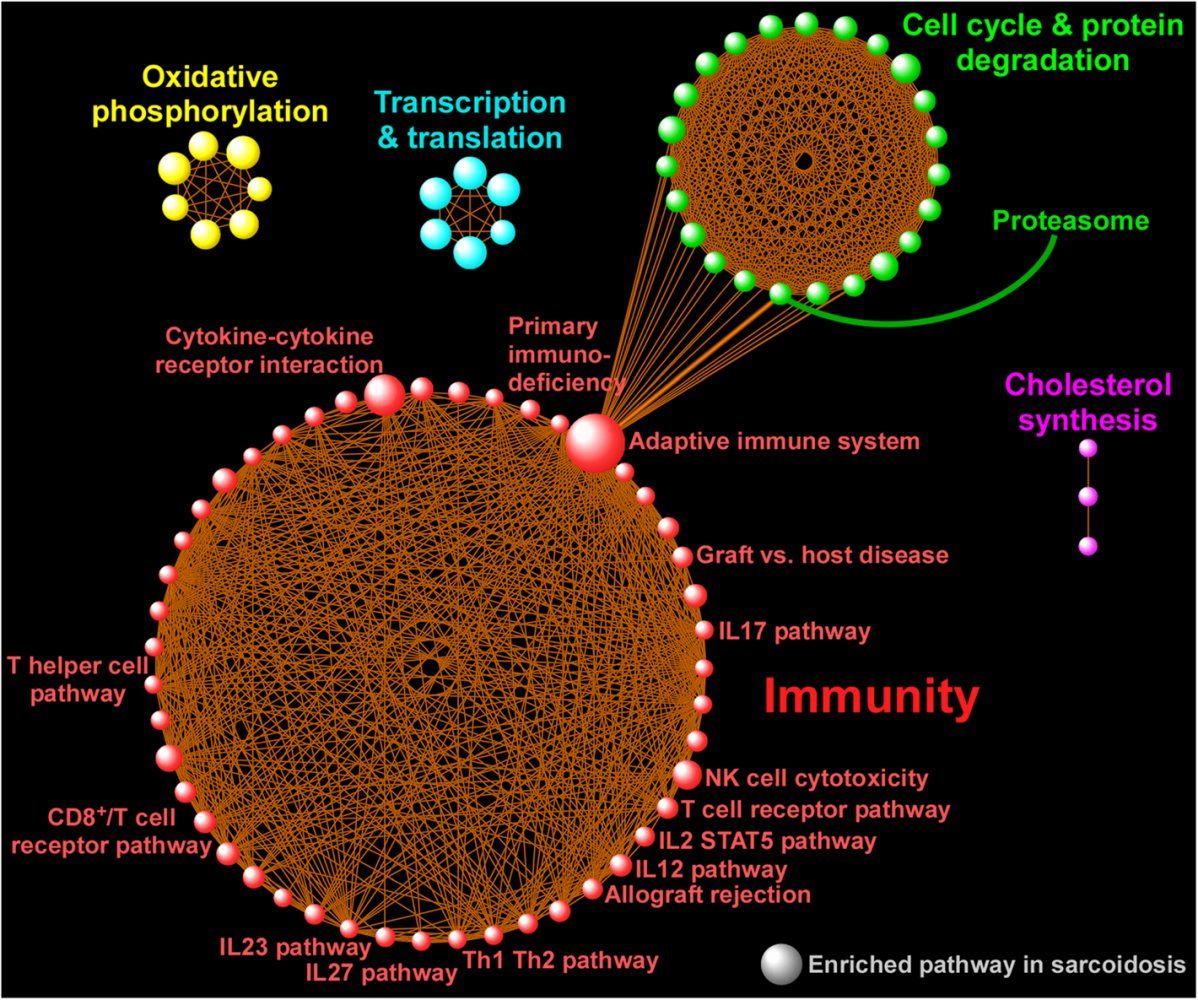
Integrative network analysis of enriched pathways in sarcoidosis as identified using GSEA. A gene set was considered enriched for a given phenotype if most of its member genes were upregulated in that condition. 83 gene sets were upregulated in patients with sarcoidosis whereas very few pathways were enriched in the controls (not shown). In the figure, each sphere designates an enriched pathway in sarcoidosis and the size of each sphere (i.e., gene set) is proportional to the number of its gene members. Only a selected number of pathways have been labeled due to space restriction, but full list is available at Additional file 3: Table S2. Since pathways can share common genes, connectivity lines have been drawn to link these inter-pathway relationships and define the topology of the enrichment network by identifying larger aggregates of functionally associated pathways known as modules. The most prominent module associated with sarcoidosis was “immunity” and incorporated T-cell signaling (T helper and CD8), graft vs. host disease, IL12, IL23, and IL17 pathways among others, but also note the presence of other modules such as “oxidative phosphorylation” and “cell cycle/protein degradation”. Importantly, some pathways from different modules were also linked together, such as the inter-modular connectivity of “adaptive immune system” with “proteasome” as shown in the figure. This relationship indicates that these two gene sets share common functional characteristics and implicates proteasomal processes as potential modulators of adaptive immunity in sarcoidosis

To obtain a more comprehensive and detailed survey of activated pathways in patients with sarcoidosis, we applied GSEA to the entire BAL cell microarray dataset. We interrogated over 1300 manually curated canonical pathways and identified 86 gene sets that were significantly enriched in sarcoidosis (FDR < 1 %), but found no significant enrichment in the control group (Additional file 3: Table S2). This observation indicated that sarcoidosis is associated with upregulation of many distinct pathways in BAL cells. We constructed a gene set enrichment network whose members (nodes) were upregulated pathways and their connectivity was based on commonly shared genes (Fig. 3). We observed that functionally similar pathways aggregated into larger groups known as modules. There is increasing recognition that biologic modules are critical drivers of disease susceptibility and progression [19–21]. The most prominent module associated with sarcoidosis was comprised of multiple processes involved in immunity, with the largest pathway being “adaptive immune system” (Fig. 3). Many of this module’s immunologic pathways have been implicated in the pathogenesis of sarcoidosis including aberrant T cell signaling, the IL17 pathway [22] as well as IL12, IL27, and IL23 family of cytokines [23, 24]. Interestingly, natural killer (NK) cell cytotoxicity was also a member of this module, and deficiency in NK cell function has been linked to progression of sarcoidosis [25–27].

**Fig. 4:**
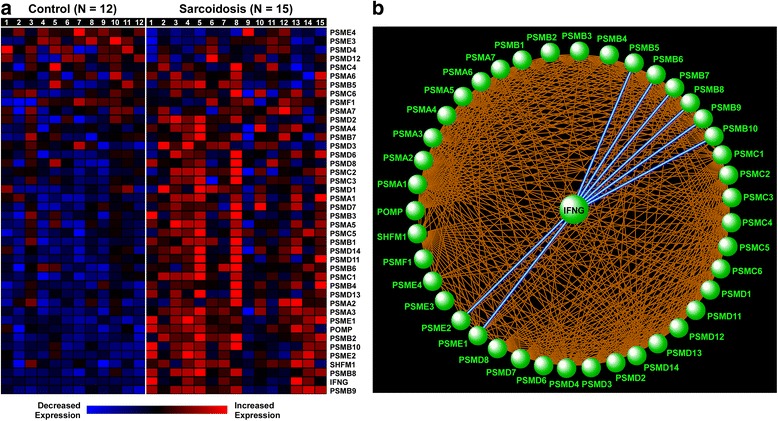
Heatmap display (**a**) and gene product interaction network (**b**) of the enriched proteasome pathway in sarcoidosis. Subject numbers correspond to Table 1 descriptions. Note the prominent role of IFNG in integrating key immunoproteasome components, such as PSMB8 and PSMB9

Another large module was highlighted by cell cycle and protein degradation processes, including the “proteasome” pathway (Fig. 3). The proteasomal system is a protein-destroying apparatus that is critical for many basic cellular functions such as cell cycle, differentiation, apoptosis, and immune-related processes such as antigen processing and presentation [28]. Most members of the proteasome pathway were upregulated in patients with sarcoidosis relative to control subjects (Fig. 4a) and formed a tightly linked interactome based on network analysis of experimentally verified, direct gene product interaction databases (Fig. 4b). This analysis highlighted the relationship between IFNG and several proteasomes (e.g., PSMB8, PSMB9) that are integral components of the immunoproteasome—the key proteolytic machinery for MHC1 antigen presentation [29]. Indeed, as shown in Fig. 3, the “proteasome” pathway is connected to “adaptive immune system” indicating that these processes share common genes and overlapping functions. Collectively, these findings reveal that sarcoidosis alters specific, highly interactive immune and proteasomal pathways in BAL cells.

### Comparative analysis of BAL cell transcriptome versus lung tissue and circulating leukocyte gene expression profiles in sarcoidosis

To extend and compare our findings to previous important work in this field, we obtained publicly available microarray data from two published studies (http://www.ncbi.nlm.nih.gov/geo, GSE16538, GSE19314): 1) lung biopsy tissue from patients with sarcoidosis (*N* = 6) and control subjects (*N* = 6) as reported by Crouser et al. [30]; 2) peripheral blood leukocyte (PBL) gene expression in sarcoidosis (*N* = 38) and healthy subjects (*N* = 20) from Koth et al. [31]. Both studies used the Affymetrix Human GeneChip U133 Plus 2.0 microarray platform. We processed each dataset via the same pipeline used for our BAL cell transcriptional analysis, including background adjustment and quantile normalization using RMA followed by pathway analysis with GSEA.

Using an FDR < 1 % cutoff to designate significant enrichment, we identified 32 gene sets upregulated in lung tissue and 47 gene sets upregulated in PBLs of patients with sarcoidosis (Additional file 4: Table S3, Additional file 5: Table S4). As summarized in Table 2, we found broad functional overlap between BAL cell and lung tissue pathway enrichment, but surprisingly few common gene sets between either lung compartment (BAL cell, tissue) versus circulating leukocytes in sarcoidosis patients. Our comparative analysis highlights a number of potentially important messages. Firstly, GSEA of BAL cells identified a substantially larger repertoire of activated pathways in sarcoidosis patients (*N* = 86 gene sets) compared to lung tissue (*N* = 32) or PBLs (*N* = 47). This finding suggests that airspace immune effector cells may provide a more robust and consistent signal compared to the more heterogeneous cell types obtained from lung tissue biopsies or the lack of proximal association with involved end organ as is the case with circulating leukocytes. Secondly, we found that in both BAL cells and lung tissue biopsies of sarcoidosis patients, T cell-associated gene sets were significantly upregulated but in contrast, these pathways were downregulated in the PBL dataset. While the implications of this observation are not clear, the known critical role played by of activation of Th1 and T cell receptor signaling in the pathobiology of sarcoidosis highlights the importance of sampling cells from affected target organs to understand mechanisms of disease pathogenesis. Finally, the enrichment of the proteasome pathway in sarcoidosis was found only in our BAL cell analysis. Given the importance of this molecular machinery in modulating adaptive immunity and thereby having a potentially novel role in sarcoidosis, our approach of interrogating immune effector cells in the airspaces shows promise for helping unravel previously unrecognized processes in this complex disease.

**Table 2:** Summary of common and discordant enrichment of pathways between BAL cell, lung tissue and PBLs in subjects with sarcoidosis

Selective list of pathways upregulated in both BAL cell and lung tissue biopsies of sarcoidosis patients	
Graft vs. host disease	
T cell receptor signaling	
Allograft rejection	
Primary immunodeficiency	
Natural killer cell mediated cytotoxicity	
Csk pathway (activation of Csk by cAMP-dependent protein kinase inhibits signaling through T cell receptor)	
Immunoregulatory interaction between lymphoid and non lymphoid cell	
IL12 pathway	
TCRA pathway (Lck and Fyn tyrosine kinases in initiation of T cell receptor activation)	
Th1 Th2 pathway	
CTL pathway (cytotoxic T lymphocyte mediated immune response against target cells)	
NKT (natural killer T cell) pathway	
Autoimmune thyroid disease	
CTLA4 (cytotoxic T-lymphocyte-associated protein 4) pathway	
PD-1 (programmed death-1) signaling	
Type 1 diabetes mellitus	
Pathways upregulated in both BAL cell and PBLs of sarcoidosis patients	
Antigen processing cross presentation	
RIP (receptor-interacting protein)-mediated NFkB activation via DAI (DNA-dependent activator of interferon)	
Pathways upregulated in BAL cell and lung tissue but downregulated in PBLs of sarcoidosis patients	
T cell receptor pathway	
Csk pathway (activation of Csk by cAMP-dependent protein kinase inhibits signaling through T cell receptor)	
TCRA pathway (Lck and Fyn tyrosine kinases in initiation of T cell receptor activation)	
CTLA4 (cytotoxic T-lymphocyte-associated protein 4) pathway	

### Confirmation of select candidate genes

**Fig. 5:**
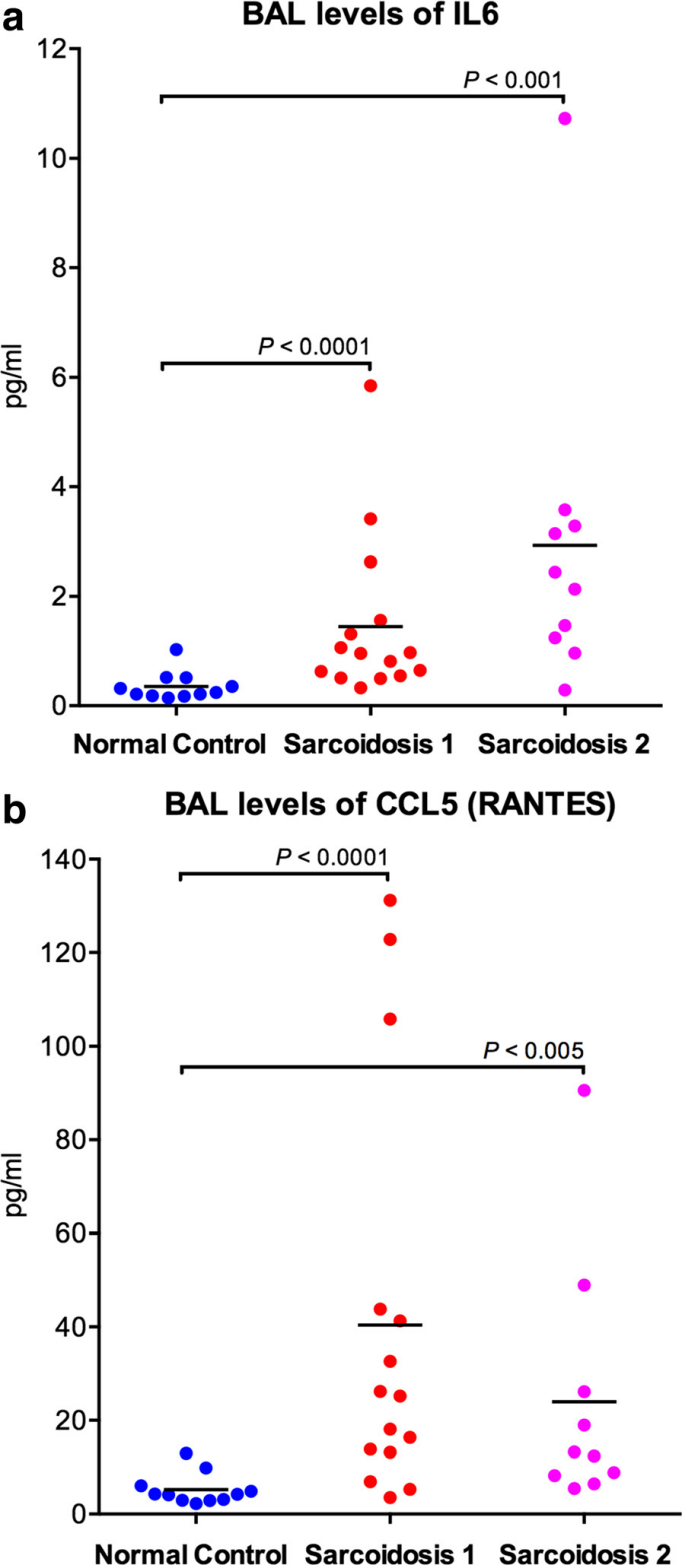
BAL levels of IL6 (**a**) and CCL5 (**b**) are elevated in sarcoidosis patients. Cell-free bronchoalveolar fluid (BALF) was analyzed by Mesoscale Discovery in normal controls (*N* = 12), sarcoidosis subjects who underwent the microarray analysis of their BAL cells (sarcoidosis 1, *N* = 15) and a validation group of sarcoidosis patients that were not part of the original microarray study (sarcoidosis 2, *N* = 10). *P*-values were calculated using two-tailed Mann–Whitney test

**Fig. 6:**
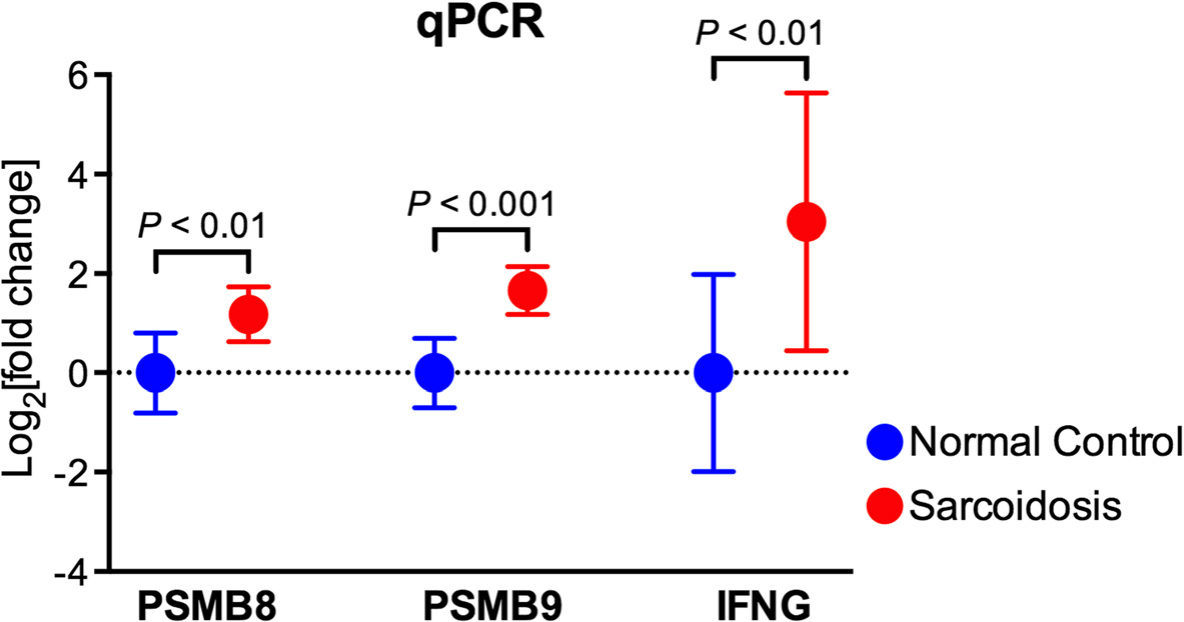
Immunoproteasome components are overexpressed in BAL cells from patients with sarcoidosis. PSMB8, PSMB9, and IFNG gene expression levels were determined by qPCR from RNA isolated from BAL cells of normal controls and sarcoidosis patients. RNA expression was normalized to GAPDH as described in Methods section. Data are presented as Log_2_ [fold change] in mRNA expression between sarcoidosis patients versus control subjects. *P*-values were calculated using two-tailed Student’s *t*-test of log_2_-transformed data

We assessed whether the immuno-inflammatory signal observed in BAL cells of patients with sarcoidosis was also present in their BAL fluid by measuring protein levels of two differentially upregulated genes: IL6 and CCL5. As shown in Fig. 5, BAL fluid levels of both cytokines were significantly elevated in the initial cohort of patients from the microarray study (sarcoidosis 1, *N* = 15) as well as an independent group of subjects with sarcoidosis (sarcoidosis 2, *N* = 10). Subject characteristics of the independent sarcoidosis group are available at Additional file 6: Table S5. Since our computational analyses associated the activation of proteasome pathway with adaptive immunity, we validated the differential upregulation of two important immunoproteasomes (PSMB8, PSMB9) as well as IFNG, the critical link between immune response and proteasomal apparatus, in BAL cells using qPCR (Fig. 6).

## Discussion

The etiology of sarcoidosis continues to be debated, with much investigation into potential instigators of the observed exaggerated and aberrant immune response. Since the lung is the most common site of organ involvement, we hypothesized that investigating the airspace immune cell transcriptome would provide insight into disease pathogenesis and reveal putative novel therapeutic targets.

We compared the transcriptional profiles of lung BAL cells in sarcoidosis patients versus control subjects and found significant differences between the two groups, most prominently the selective enrichment of distinct immune-related pathways. As confirmation of our unbiased approach, we identified a number of immune modulators previously implicated in sarcoidosis, including IFNG [32], IL12 [23], and IL17 [22]. Notably, in addition to observing enrichment of T helper cell signaling that is well-described in sarcoidosis [33], we also found strong signals from CD8/T cell receptor pathways, potentially implicating a role for autoimmune T cells in sustaining inflammatory responses and contributing to the pathogenesis of this complex disease [34].

Our data are consistent with studies of whole blood and lung tissue transcriptome which demonstrated upregulation of interferon signaling and activation of immuno-inflammatory pathways [30–32, 35]. Similarly, some of our findings overlap with gene expression analysis of sarcoidosis skin lesions that also showed involvement of IL23 and IL17 signaling [36]. Collectively, these studies outline a system-wide transcriptional signature associated with sarcoidosis that may be useful for diagnostic purposes. However, we also found significant differences in gene set enrichment across different compartments (BAL cell, lung tissue, circulating leukocytes) consistent with the concept that host response in sarcoidosis is cell-type and organ specific.

By integrating pathway and network analysis of BAL cell expression profiles, we constructed a modular overview of differentially activated processes in sarcoidosis that linked key putative drivers of disease. In particular, our analysis highlighted a potentially important connection between adaptive immunity and the proteasomal apparatus. The proteasome is part of the ubiquitin-proteasome system, which controls intracellular protein degradation and turnover. Activation of proteasome complex components suggests that sarcoidosis induces a proteolytic environment for substrate degradation. We found upregulation of constitutive proteasome catalytic subunits and the inducible proteasome subunits, PSMB8, PSMB9 and PSMB10. The inducible subunits form the immunoproteasome, which is upregulated by oxidative stress and proinflammatory cytokines, particularly IFNG [37]. Key functions of the immunoproteasome are processing class I MHC peptides and regulation of inflammation, which are of particular relevance to the pathogenesis of sarcoidosis. The immunoproteasome regulates inflammatory responses through the activation of NFkB, which then induces expression of proinflammatory cytokines such as tumor necrosis factor (TNF), interleukin 1 beta (IL1B), and interleukin 8 (IL8) [38]. The proteasome machinery can also proteolyze cyclins which impacts the inflammatory responses through regulation of leukocyte proliferation [39]. Consistent with this role, our modular enrichment network demonstrated tight linkage between the proteasome and cell cycle regulators.

Our findings are consistent with a recent report by Sixt et al. [40], who found significantly higher levels of extracellular BAL proteasome activity in sarcoidosis patients. They demonstrated increased immunoreactivity of PSMB8 and PSMB9 in sarcoidosis lungs, primarily in epithelial cells and granulomas [40]. Our data confirm and expand their findings by showing that multiple components of proteasome are upregulated in the lung immune effector cells and that these processes may be linked to immune and cell cycle pathways via specific network hubs such as IFNG. While prior transcriptomic studies of lung tissue and blood did not identify enrichment of proteasome components, all reported upregulation of IFNG, a key regulator of the immunoproteasome [30–32, 35]. Importantly, proteasomal pathways are high priority targets for a new class of therapeutic drugs in a variety of diseases including cancer, neurodegenerative disorders, and autoimmune diseases [41–43]. Future work is needed to assess the intriguing possibility that these drugs may be effective for treatment of sarcoidosis.

Our study has a number of limitations. Since the number of subjects was relatively small, our analyses should be considered exploratory and require confirmation in larger cohorts. However, our sample size was similar to previous reports leveraging tissue gene expression profiling to elucidate disease mechanisms in sarcoidosis [30, 36]. Since only two patients were on systemic steroids, we were unable to determine an effect of therapy on gene expression signatures. Our study was cross-sectional, therefore longitudinal follow-up studies are needed to establish whether any of the transcriptional signatures correlate with disease progression. There was significant heterogeneity among the sarcoidosis patients, however, we did not observe distinct segregation based on race or disease severity when applying cluster analysis to the BAL cell expression data. Furthermore, a number of the sarcoidosis patients undergoing gene expression analysis were former smokers, a potential confounding environmental exposure [44]. Although we did not separate out the different lung cell populations, the most dominant cell type in the subject cohort was alveolar macrophages. However, several sarcoidosis patients also had lymphocytosis and these cells likely contributed to overall gene expression. Nevertheless, we did not observe segregation of sarcoidosis patients based on their relative BAL lymphocyte or macrophage counts when applying cluster analysis (Fig. 2), suggesting that variability in cell count percentages did not heavily influence the overall transcriptional signal. Future studies investigating cell-specific gene expression of BAL cells (e.g., lymphocytes and macrophages) will be important to further delineate the selective role of immune cell subtypes in sarcoidosis. By design, all study patients had pulmonary sarcoidosis. It will be of interest to determine whether the lung cell transcriptome in sarcoidosis patients without overt lung involvement has similar expression patterns. Our comparative analysis using available gene expression profiles from other tissues and compartments supports this concept and suggests that sarcoidosis is associated with perturbation in a common core of pathways spanning target end organs.

## Conclusion

We have outlined a systematic framework to investigate the role of airspace immune cells in the pathogenesis of sarcoidosis. We found that this complex disease is associated with the activation of diverse yet distinct pathways and identified key members of the proteasome apparatus as potential modulators of host immune response. Future work is required to further delineate these relationships and determine their influence in disease progression.

## Additional files

Additional file 1: Figure S1. Overview of Analytic Approach (PDF 1072 kb) Additional file 2: Table S1. Detailed subject demographics and BAL cell counts. (PDF 358 kb) Additional file 3: Table S2. List of significantly upregulated gene sets in BAL cells of sarcoidosis patients. Note that no gene sets were downregulated at the FDR < 1 % threshold. (PDF 183 kb) Additional file 4: Table S3. List of significantly upregulated and downregulated gene sets in lung tissue of sarcoidosis patients at FDR < 1 % threshold. (PDF 101 kb) Additional file 5: Table S4. List of significantly upregulated and downregulated gene sets in PBLs of sarcoidosis patients at FDR < 1 % threshold. Note that several T cell--‐associated pathways were downregulated in PBLs of sarcoidosis patients. (PDF 278 kb) Additional file 6: Table S5. Subject demographics and BAL differential cell counts (sarcoidosis 2). (PDF 117 kb)
