# Supplementary material for: Sarcoidosis activates diverse transcriptional programs in bronchoalveolar lavage cells
Source: Respir Res. 2016 Jul 26;17:93. doi: 10.1186/s12931-016-0411-y (PMC4962428; doi:10.1186/s12931-016-0411-y)

Overview of Analytic Approach for Pathway Enrichment and Network Analysis in Sarcoidosis

Control (N = 12)

Sarcoidosis (N = 15)

GSEA:
relative enrichment of ~ 1300 canonical pathways between control and sarcoidosis subjects statistically evaluated

Enrichment Map:
GSEA map displays relationships among enriched (up-regulated) pathways in sarcoidosis patients relative to controls

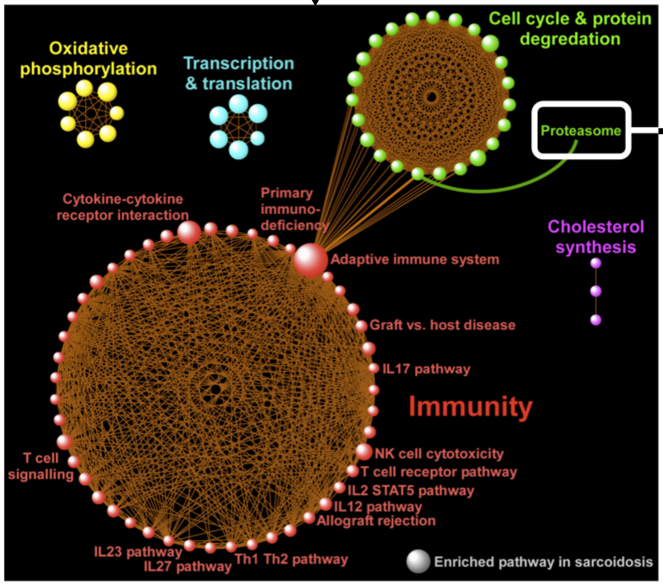

Network Analysis:
Connectivity between gene members of Proteasome, an enriched pathway in sarcoidosis, was determined based on previously published gene product interactions obtained from available resources including Ingenuity and STRING

Proteasome Network

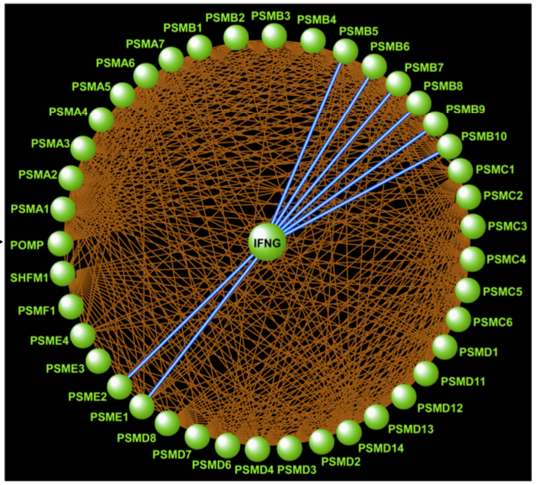

Supplement: Additional file 1: Figure S1. — Overview of Analytic Approach (PDF 1072 kb) [file 12931_2016_411_MOESM1_ESM.pdf]
