# Supplementary material for: Sarcoidosis activates diverse transcriptional programs in bronchoalveolar lavage cells
Source: Respir Res. 2016 Jul 26;17:93. doi: 10.1186/s12931-016-0411-y (PMC4962428; doi:10.1186/s12931-016-0411-y)
Supplement: Additional file 2: Table S1. — Detailed subject demographics and BAL cell counts. (PDF 358 kb) [file 12931_2016_411_MOESM2_ESM.pdf]

Additional file 2: Table S1 Detailed subject demographics and BAL cell counts

Subject	Gender	Age	Race	Smoking	Stage	Mac (%)	Lymph (%)	Pmn (%)	Eos (%)	FVC (%)	Affected organs	Oral steroid at time of bronch
Sarc 1	M	52	AA	Former	3	68	32	0	0	79	skin, lungs	No
Sarc 2	F	32	AA	Former	3	67	30	3	0	77	lungs	No
Sarc 3	F	39	AA	Never	2	89	10	1	0	60	LN's, lungs	No
Sarc 4	F	61	AA	Never	NA	64	35	1	0	68	LN's, lungs	No
Sarc 5	M	57	AA	Former	3	95	4	0	1	56	lungs	No
Sarc 6	M	68	AA	Never	4	86	14	0	0	107	LN's, lungs	No
Sarc 7	F	62	AA	Never	0	53	47	0	0	85	lung, skin	No
Sarc 8	M	34	AA	Never	2	91	8	1	0	77	skin, lungs, LN	No
Sarc 9	F	47	AA	Former	4	97	3	0	0	93	lungs, liver, spleen, lungs	No
Sarc 10	M	48	AA	Never	2	61	37	2	0	NA	lung, skin	No
Sarc 11	F	53	C	Never	2	96	4	0	0	93	lungs, LN,joints	No
Sarc 12	F	39	AA	Never	2	70	30	0	0	56	lungs, cardiac, skin	No
Sarc 13	F	42	AA	Former	1	85	15	0	0	63	LN's, lungs	No
Sarc 14	F	46	AA	Never	2	67	9	24	0	NA	lungs	Yes
Sarc 15	M	49	AA	Former	2	94	5	0	1	66	lungs, LN's	Yes
Cont 1	M	27	AA	Never	__	92	5	3	0	__	__	__
Cont 2	M	28	AA	Never	__	98	2	0	0	__	__	__
Cont 3	F	51	C	Never	__	92	8	0	0	__	__	__
Cont 4	F	31	AA	Never	__	92	8	0	0	__	__	__
Cont 5	F	26	AA	Never	__	91	4	3	2	__	__	__
Cont 6	F	24	AA	Never	__	95	5	0	0	__	__	__
Cont 7	F	28	AA	Never	__	98	1	0	1	__	__	__
Cont 8	M	28	C	Never	__	98	2	0	0	__	__	__
Cont 9	F	26	AA	Never	__	98	2	0	0	__	__	__
Cont 10	F	27	AA	Never	__	97	3	0	0	__	__	__
Cont 11	M	30	AA	Never	__	95	5	0	0	__	__	__
Cont 12	F	35	AA	Never	__	93	7	0	0	__	__	__
