# Supplementary material for: Sarcoidosis activates diverse transcriptional programs in bronchoalveolar lavage cells
Source: Respir Res. 2016 Jul 26;17:93. doi: 10.1186/s12931-016-0411-y (PMC4962428; doi:10.1186/s12931-016-0411-y)
Supplement: Additional file 3: Table S2. — List of significantly upregulated gene sets in BAL cells of sarcoidosis patients. Note that no gene sets were downregulated at the FDR < 1 % threshold. (PDF 183 kb) [file 12931_2016_411_MOESM3_ESM.pdf]

**Additiona file 3: Table S2** List of significantly upregulated gene sets in BAL cells of sarcoidosis patients.
Note that no gene sets were downregulated at the FDR < 1% threshold.

| Upregulated gene set | Number of genes | FDR |
|---|---|---|
| PID_IL12_2PATHWAY | 62 | 0 |
| REACTOME_RESPIRATORY_ELECTRON_TRANSPORT_ATP_SYNTHESIS_BY_CHEMIOSMOTIC_COUPLING_AND_HEAT_PRODUCTION | 74 | 0 |
| REACTOME_RESPIRATORY_ELECTRON_TRANSPORT | 58 | 0 |
| PID_CD8TCRPATHWAY | 49 | 0 |
| BIOCARTA_NO2IL12_PATHWAY | 17 | 0 |
| KEGG_PARKINSONS_DISEASE | 102 | 0 |
| PID_CD8TCRDOWNSTREAMPATHWAY | 62 | 0 |
| KEGG_OXIDATIVE_PHOSPHORYLATION | 103 | 0 |
| REACTOME_IMMUNOREGULATORY_INTERACTIONS_BETWEEN_A_LYMPHOID_AND_A_NON_LYMPHOID_CELL | 58 | 0 |
| KEGG_ALLOGRAFT_REJECTION | 34 | 0 |
| REACTOME_TCA_CYCLE_AND_RESPIRATORY_ELECTRON_TRANSPORT | 107 | 0 |
| BIOCARTA_IL12_PATHWAY | 21 | 0 |
| REACTOME_ER_PHAGOSOME_PATHWAY | 57 | 0 |
| REACTOME_CHOLESTEROL_BIOSYNTHESIS | 20 | 0 |
| KEGG_GRAFT_VERSUS_HOST_DISEASE | 37 | 0 |
| ST_T_CELL_SIGNAL_TRANSDUCTION | 43 | 0 |
| BIOCARTA_NKT_PATHWAY | 29 | 0 |
| REACTOME_CHEMOKINE_RECEPTORS_BIND_CHEMOKINES | 50 | 0 |
| BIOCARTA_TCRA_PATHWAY | 11 | 0 |
| REACTOME_TRANSLOCATION_OF_ZAP_70_TO_IMMUNOLOGICAL_SYNAPSE | 11 | 0 |
| KEGG_CYTOKINE_CYTOKINE_RECEPTOR_INTERACTION | 237 | 0 |
| REACTOME_TCR_SIGNALING | 47 | 0 |
| KEGG_RIBOSOME | 86 | 0.0001 |
| BIOCARTA_TOB1_PATHWAY | 19 | 0.0001 |
| REACTOME_GENERATION_OF_SECOND_MESSENGER_MOLECULES | 24 | 0.0001 |
| REACTOME_PEPTIDE_CHAIN_ELONGATION | 85 | 0.0001 |
| KEGG_AUTOIMMUNE_THYROID_DISEASE | 48 | 0.0001 |
| REACTOME_APC_C_CDC20_MEDIATED_DEGRADATION_OF_MITOTIC_PROTEINS | 60 | 0.0001 |
| PID_IL23PATHWAY | 35 | 0.0001 |
| REACTOME_SRP_DEPENDENT_COTRANSLATIONAL_PROTEIN_TARGETING_TO_MEMBRANE | 106 | 0.0001 |
| REACTOME_AUTODEGRADATION_OF_CDH1_BY_CDH1_APC_C | 52 | 0.0001 |
| PID_IL12_STAT4PATHWAY | 33 | 0.0001 |
| REACTOME_PHOSPHORYLATION_OF_CD3_AND_TCR_ZETA_CHAINS | 12 | 0.0001 |
| PID_NFAT_TFPATHWAY | 45 | 0.0001 |
| BIOCARTA_TH1TH2_PATHWAY | 19 | 0.0001 |
| KEGG_T_CELL_RECEPTOR_SIGNALING_PATHWAY | 104 | 0.0002 |
| KEGG_STEROID_BIOSYNTHESIS | 16 | 0.0002 |
| REACTOME_SCFSKP2_MEDIATED_DEGRADATION_OF_P27_P21 | 53 | 0.0002 |
| REACTOME_VIF_MEDIATED_DEGRADATION_OF_APOBEC3G | 48 | 0.0002 |
| KEGG_PRIMARY_IMMUNODEFICIENCY | 34 | 0.0002 |
| BIOCARTA_TCYTOTOXIC_PATHWAY | 12 | 0.0002 |
| PID_TCR_PATHWAY | 60 | 0.0002 |
| REACTOME_REGULATION_OF_MITOTIC_CELL_CYCLE | 72 | 0.0003 |
| BIOCARTA_CTL_PATHWAY | 13 | 0.0003 |
| KEGG_NATURAL_KILLER_CELL_MEDIATED_CYTOTOXICITY | 123 | 0.0003 |
| REACTOME_CYCLIN_E_ASSOCIATED_EVENTS_DURING_G1_S_TRANSITION_ | 62 | 0.0004 |
| BIOCARTA_TCAPOPTOSIS_PATHWAY | 9 | 0.0004 |
| REACTOME_APC_C_CDH1_MEDIATED_DEGRADATION_OF_CDC20_AND_OTHER_APC_C_CDH1_TARGETED_PROTEINS_IN_LATE_MITOSIS_EARLY_G1 | 59 | 0.0006 |
| BIOCARTA_CTLA4_PATHWAY | 19 | 0.0006 |
| KEGG_PROTEASOME | 42 | 0.0006 |
| KEGG_INTESTINAL_IMMUNE_NETWORK_FOR_IGA_PRODUCTION | 42 | 0.0007 |
| PID_IL2_STAT5PATHWAY | 30 | 0.0008 |
| REACTOME_INFLUENZA_VIRAL_RNA_TRANSCRIPTION_AND_REPLICATION | 101 | 0.0010 |
| BIOCARTA_ASBCELL_PATHWAY | 12 | 0.0010 |
| REACTOME_SCF_BETA_TRCP_MEDIATED_DEGRADATION_OF_EMI1 | 48 | 0.0014 |
| REACTOME_REGULATION_OF_ORNITHINE_DECARBOXYLASE_ODC | 47 | 0.0015 |
| BIOCARTA_TCR_PATHWAY | 43 | 0.0016 |
| KEGG_ALZHEIMERS_DISEASE | 147 | 0.0020 |
| BIOCARTA_STATHMIN_PATHWAY | 18 | 0.0021 |
| REACTOME_CDK_MEDIATED_PHOSPHORYLATION_AND_REMOVAL_OF_CDC6 | 45 | 0.0021 |
| BIOCARTA_CSK_PATHWAY | 22 | 0.0023 |
| BIOCARTA_THELPER_PATHWAY | 12 | 0.0027 |
| REACTOME_CDT1_ASSOCIATION_WITH_THE_CDC6_ORC_ORIGIN_COMPLEX | 52 | 0.0027 |
| REACTOME_G1_S_TRANSITION | 105 | 0.0028 |

| | | |
|---|---|---|
| BIOCARTA_IL17_PATHWAY | 15 | 0.0028 |
| KEGG_TERPENOID_BACKBONE_BIOSYNTHESIS | 14 | 0.0034 |
| REACTOME_DOWNSTREAM_TCR_SIGNALING | 31 | 0.0034 |
| KEGG_TYPE_I_DIABETES_MELLITUS | 40 | 0.0036 |
| REACTOME_ANTIGEN_PROCESSING_CROSS_PRESENTATION | 71 | 0.0036 |
| REACTOME_CELL_CYCLE_CHECKPOINTS | 103 | 0.0040 |
| REACTOME_PD1_SIGNALING | 14 | 0.0042 |
| REACTOME_ADAPTIVE_IMMUNE_SYSTEM | 453 | 0.0047 |
| REACTOME_DESTABILIZATION_OF_MRNA_BY_AUF1_HNRNP_D0 | 48 | 0.0053 |
| REACTOME_RIP_MEDIATED_NFKB_ACTIVATION_VIA_DAI | 17 | 0.0062 |
| PID_IL27PATHWAY | 25 | 0.0069 |
| REACTOME_AUTODEGRADATION_OF_THE_E3_UBIQUITIN_LIGASE_COP1 | 45 | 0.0069 |
| REACTOME_ORC1_REMOVAL_FROM_CHROMATIN | 63 | 0.0070 |
| REACTOME_M_G1_TRANSITION | 76 | 0.0070 |
| KEGG_HEMATOPOIETIC_CELL_LINEAGE | 85 | 0.0072 |
| REACTOME_3_UTR_MEDIATED_TRANSLATIONAL_REGULATION | 105 | 0.0074 |
| REACTOME_P53_INDEPENDENT_G1_S_DNA_DAMAGE_CHECKPOINT | 47 | 0.0077 |
| REACTOME_P53_DEPENDENT_G1_DNA_DAMAGE_RESPONSE | 51 | 0.0087 |
| KEGG_HUNTINGTONS_DISEASE | 156 | 0.0092 |
| REACTOME_MITOTIC_G1_G1_S_PHASES | 126 | 0.0097 |
| REACTOME_NONSENSE_MEDIATED_DECAY_ENHANCED_BY_THE_EXON_JUNCTION_COMPLEX | 104 | 0.0098 |
| REACTOME_THE_ROLE_OF_NEF_IN_HIV1_REPLICATION_AND_DISEASE_PATHOGENESIS | 25 | 0.0099 |
