# Supplementary material for: Sarcoidosis activates diverse transcriptional programs in bronchoalveolar lavage cells
Source: Respir Res. 2016 Jul 26;17:93. doi: 10.1186/s12931-016-0411-y (PMC4962428; doi:10.1186/s12931-016-0411-y)
Supplement: Additional file 4: Table S3. — List of significantly upregulated and downregulated gene sets in lung tissue of sarcoidosis patients at FDR < 1 % threshold. (PDF 101 kb) [file 12931_2016_411_MOESM4_ESM.pdf]

Additiona file 4: Table S3 List of significantly upregulated and downregulated gene sets in lung tissue of sarcoidosis patients at FDR < 1% threshold.

Upregulated gene set	Number of genes	FDR
KEGG_GRAFT_VERSUS_HOST_DISEASE	37	0
REACTOME_TCR_SIGNALING	49	0
REACTOME_GENERATION_OF_SECOND_MESSENGER_MOLECULES	24	0
BIOCARTA_CSK_PATHWAY	22	0.0005
BIOCARTA_IL7_PATHWAY	17	0.0006
KEGG_TYPE_I_DIABETES_MELLITUS	40	0.0008
KEGG_ALLOGRAFT_REJECTION	34	0.0008
REACTOME_INTERFERON_GAMMA_SIGNALING	56	0.0011
KEGG_PRIMARY_IMMUNODEFICIENCY	35	0.0017
REACTOME_IMMUNOREGULATORY_INTERACTIONS_BETWEEN_A_LYMPHOID_AND_A_NON_LYMPHOID_CELL	61	0.0025
KEGG_NATURAL_KILLER_CELL_MEDIATED_CYTOTOXICITY	130	0.0026
REACTOME_PHOSPHORYLATION_OF_CD3_AND_TCR_ZETA_CHAINS	13	0.0026
PID_IL12_2PATHWAY	62	0.0033
KEGG_AUTOIMMUNE_THYROID_DISEASE	48	0.0034
REACTOME_INTERFERON_SIGNALING	149	0.0035
BIOCARTA_TCRA_PATHWAY	11	0.0041
REACTOME_PD1_SIGNALING	15	0.0042
REACTOME_ANTIGEN_ACTIVATES_B_CELL_RECEPTOR_LEADING_TO_GENERATION_OF_SECOND_MESSENGERS	27	0.0043
REACTOME_GENERIC_TRANSCRIPTION_PATHWAY	327	0.0046
KEGG_ANTIGEN_PROCESSING_AND_PRESENTATION	81	0.0046
PID_CD8_TCR_DOWNSTREAM_PATHWAY	65	0.0052
BIOCARTA_NKT_PATHWAY	29	0.0058
REACTOME_REGULATION_OF_KIT_SIGNALING	16	0.0058
REACTOME_SIGNALING_BY_BMP	22	0.0060
BIOCARTA_CTL_PATHWAY	13	0.0068
BIOCARTA_TH1TH2_PATHWAY	19	0.0068
REACTOME_CYTOKINE_SIGNALING_IN_IMMUNE_SYSTEM	258	0.0071
BIOCARTA_CTCF_PATHWAY	23	0.0072
REACTOME_TRANSLOCATION_OF_ZAP_70_TO_IMMUNOLOGICAL_SYNAPSE	11	0.0076
BIOCARTA_CTLA4_PATHWAY	19	0.0087
REACTOME_DOWNSTREAM_TCR_SIGNALING	32	0.0088
BIOCARTA_TCYTOTOXIC_PATHWAY	12	0.0093
BIOCARTA_STATHMIN_PATHWAY	19	0.0093
KEGG_B_CELL_RECEPTOR_SIGNALING_PATHWAY	73	0.0096

Downregulated gene set	Number of genes	FDR
REACTOME_CHOLESTEROL_BIOSYNTHESIS	21	0
REACTOME_ACTIVATION_OF_CHAPERONE_GENES_BY_XBP1S	43	0.0055
