# Supplementary material for: Sarcoidosis activates diverse transcriptional programs in bronchoalveolar lavage cells
Source: Respir Res. 2016 Jul 26;17:93. doi: 10.1186/s12931-016-0411-y (PMC4962428; doi:10.1186/s12931-016-0411-y)
Supplement: Additional file 5: Table S4. — List of significantly upregulated and downregulated gene sets in PBLs of sarcoidosis patients at FDR < 1 % threshold. Note that several T cell--‐associated pathways were downregulated in PBLs of sarcoidosis patients. (PDF 278 kb) [file 12931_2016_411_MOESM5_ESM.pdf]

Additiona file 5: Table S4 List of significantly upregulated and downregulated gene sets in PBLs of sarcoidosis patients at FDR < 1% threshold.
Note that several T cell-associated pathways were downregulated in PBLs of sarcoidosis patients.

Upregulated gene set	Number of genes	FDR
REACTOME_INTERFERON_GAMMA_SIGNALING	56	0
REACTOME_INTERFERON_ALPHA_BETA_SIGNALING	62	0
REACTOME_INTERFERON_SIGNALING	149	0
REACTOME_CYTOKINE_SIGNALING_IN_IMMUNE_SYSTEM	258	0
REACTOME_INNATE_IMMUNE_SYSTEM	246	0
KEGG_LEISHMANIA_INFECTION	69	0
KEGG_NEUROACTIVE_LIGAND_RECEPTOR_INTERACTION	267	0
BIOCARTA_COMP_PATHWAY	18	0
REACTOME_COMPLEMENT_CASCADE	29	0
KEGG_COMPLEMENT_AND_COAGULATION_CASCADES	68	0
REACTOME_ACTIVATED_TAK1_MEDIATES_P38_MAPK_ACTIVATION	16	0
REACTOME_EICOSANOID_LIGAND_BINDING_RECEPTORS	14	0.0003
REACTOME_TOLL_RECEPTOR_CASCADES	112	0.0006
REACTOME_ACTIVATED_TLR4_SIGNALLING	89	0.0006
REACTOME_ANTIGEN_PROCESSING_CROSS_PRESENTATION	71	0.0014
PID_UPA_UPAR_PATHWAY	40	0.0017
KEGG_TOLL_LIKE_RECEPTOR_SIGNALING_PATHWAY	100	0.0017
REACTOME_TAK1_ACTIVATES_NFKB_BY_PHOSPHORYLATION_AND_ACTIVATION_OF_IKKS_COMPLEX	23	0.0017
KEGG_NOD_LIKE_RECEPTOR_SIGNALING_PATHWAY	61	0.0017
REACTOME_TRIF_MEDIATED_TLR3_SIGNALING	71	0.0018
KEGG_SYSTEMIC_LUPUS_ERYTHEMATOSUS	90	0.0018
PID_IFNG_PATHWAY	39	0.0019
REACTOME_MYD88_MAL_CASCADE_INITIATED_ON_PLASMA_MEMBRANE	79	0.0019
BIOCARTA_CLASSIC_PATHWAY	13	0.0019
REACTOME_IL1_SIGNALING	38	0.0019
REACTOME_TRAF6_MEDIATED_INDUCTION_OF_NFKB_AND_MAP_KINASES_UPON_TLR7_8_OR_9_ACTIVATION	73	0.0025
KEGG_NOTCH_SIGNALING_PATHWAY	47	0.0029
REACTOME_REGULATION_OF_IFNA_SIGNALING	24	0.0030
PID_INTEGRIN_A9B1_PATHWAY	25	0.0030
REACTOME_ACTIVATED_NOTCH1_TRANSMITS_SIGNAL_TO_THE_NUCLEUS	26	0.0031
REACTOME_GPCR_LIGAND_BINDING	378	0.0031
REACTOME_NFKB_AND_MAP_KINASES_ACTIVATION_MEDIATED_BY_TLR4_SIGNALING_REPERTOIRE	69	0.0035
REACTOME_RIG_I_MDA5_MEDIATED_INDUCTION_OF_IFN_ALPHA_BETA_PATHWAYS	71	0.0041
REACTOME_TRAF6_MEDIATED_IRF7_ACTIVATION	30	0.0046
REACTOME_DEGRADATION_OF_THE_EXTRACELLULAR_MATRIX	29	0.0047
KEGG_DORSO_VENTRAL_AXIS_FORMATION	23	0.0053
REACTOME_SIGNALING_BY_ILS	105	0.0053
REACTOME_NUCLEOTIDE_BINDING_DOMAIN_LEUCINE_RICH_REPEAT_CONTAINING_RECEPTOR_NLR_SIGNALING_PATHWAYS	43	0.0056
REACTOME_CHONDROITIN_SULFATE_DERMATAN_SULFATE_METABOLISM	47	0.0056
REACTOME_GABA_B_RECEPTOR_ACTIVATION	38	0.0066
REACTOME_CHONDROITIN_SULFATE_BIOSYNTHESIS	19	0.0067
REACTOME_GROWTH_HORMONE_RECEPTOR_SIGNALING	24	0.0067
REACTOME_RIP_MEDIATED_NFKB_ACTIVATION_VIA_DAI	18	0.0067
REACTOME_INITIAL_TRIGGERING_OF_COMPLEMENT	13	0.0069
REACTOME_G_ALPHA_I_SIGNALLING_EVENTS	184	0.0082
REACTOME_AMYLOIDS	47	0.0085
REACTOME_EXTRACELLULAR_MATRIX_ORGANIZATION	85	0.0088

Downregulated gene set	Number of genes	FDR
REACTOME_TRANSLATION	142	0
REACTOME_3_UTR_MEDIATED_TRANSLATIONAL_REGULATION	102	0
REACTOME_SRP_DEPENDENT_COTRANSLATIONAL_PROTEIN_TARGETING_TO_MEMBRANE	105	0
REACTOME_PEPTIDE_CHAIN_ELONGATION	82	0
KEGG_RIBOSOME	84	0
REACTOME_NONSENSE_MEDIATED_DECAY_ENHANCED_BY_THE_EXON_JUNCTION_COMPLEX	102	0
REACTOME_INFLUENZA_VIRAL_RNA_TRANSCRIPTION_AND_REPLICATION	98	0
REACTOME_INFLUENZA_LIFE_CYCLE	131	0
REACTOME_METABOLISM_OF_RNA	247	0
REACTOME_ACTIVATION_OF_THE_MRNA_UPON_BINDING_OF_THE_CAP_BINDING_COMPLEX_AND_EIFS_AND_SUBSEQUENT_BINDING_TO_43S	54	0
REACTOME_FORMATION_OF_THE_TERNARY_COMPLEX_AND_SUBSEQUENTLY_THE_43S_COMPLEX	46	0
REACTOME_METABOLISM_OF_MRNA	203	0
REACTOME_PROCESSING_OF_CAPPED_INTRON_CONTAINING_PRE_MRNA	133	0
REACTOME_MRNA_PROCESSING	153	0
BIOCARTA_TCR_PATHWAY	42	0
REACTOME_METABOLISM_OF_NON_CODING_RNA	47	0

REACTOME_METABOLISM_OF_PROTEINS	408	0
REACTOME_GENERIC_TRANSCRIPTION_PATHWAY	327	0.0000
REACTOME_MITOCHONDRIAL_TRNA_AMINOACYLATION	21	0.0000
REACTOME_MRNA_SPLICING	104	0.0000
REACTOME_MITOCHONDRIAL_PROTEIN_IMPORT	47	0.0001
KEGG_RNA_POLYMERASE	26	0.0002
KEGG_PROTEIN_EXPORT	23	0.0002
REACTOME_TRNA_AMINOACYLATION	42	0.0003
KEGG_AMINOACYL_TRNA_BIOSYNTHESIS	41	0.0003
REACTOME_CLEAVAGE_OF_GROWING_TRANSCRIPT_IN_THE_TERMINATION_REGION_	40	0.0004
REACTOME_TRANSPORT_OF_MATURE_TRANSCRIPT_TO_CYTOPLASM	50	0.0004
KEGG_SPLICEOSOME	118	0.0004
REACTOME_INTERACTIONS_OF_VPR_WITH_HOST_CELLULAR_PROTEINS	32	0.0004
PID_CD8_TCR_PATHWAY	53	0.0006
KEGG_T_CELL_RECEPTOR_SIGNALING_PATHWAY	107	0.0007
REACTOME_RESPIRATORY_ELECTRON_TRANSPORT_ATP_SYNTHESIS_BY_CHEMIOSMOTIC_COUPLING_AND_HEAT_PRODUCTION_BY_UNCOUPLING_PROTEINS_	85	0.0009
BIOCARTA_TCRA_PATHWAY	11	0.0009
REACTOME_RNA_POL_II_TRANSCRIPTION	95	0.0009
REACTOME_PURINE_RIBONUCLEOSIDE_MONOPHOSPHATE_BIOSYNTHESIS	11	0.0010
KEGG_RNA_DEGRADATION	57	0.0015
REACTOME_BIOSYNTHESIS_OF_THE_N_GLYCAN_PRECURSOR_DOLICHOL_LIPID_LINKED_OLIGOSACCHARIDE_LLO_AND_TRANSFER_TO_A_NASCENT_PROTEIN	26	0.0016
REACTOME_SLBP_DEPENDENT_PROCESSING_OF_REPLICATION_DEPENDENT_HISTONE_PRE_MRNAS	11	0.0024
BIOCARTA_STATHMIN_PATHWAY	19	0.0033
REACTOME_HIV_LIFE_CYCLE	110	0.0044
REACTOME_NEP_NS2_INTERACTS_WITH_THE_CELLULAR_EXPORT_MACHINERY	27	0.0046
BIOCARTA_GPCR_PATHWAY	32	0.0046
REACTOME_MRNA_3_END_PROCESSING	31	0.0047
BIOCARTA_CTLA4_PATHWAY	19	0.0050
REACTOME_RESPIRATORY_ELECTRON_TRANSPORT	68	0.0051
REACTOME_TCA_CYCLE_AND_RESPIRATORY_ELECTRON_TRANSPORT	121	0.0052
REACTOME_GENERATION_OF_SECOND_MESSENGER_MOLECULES	24	0.0059
BIOCARTA_TOB1_PATHWAY	19	0.0059
REACTOME_TRANSPORT_OF_MATURE_MRNA_DERIVED_FROM_AN_INTRONLESS_TRANSCRIPT	32	0.0065
REACTOME_PROCESSING_OF_CAPPED_INTRONLESS_PRE_MRNA	23	0.0069
REACTOME_TCR_SIGNALING	49	0.0072
REACTOME_LATE_PHASE_OF_HIV_LIFE_CYCLE	97	0.0079
BIOCARTA_CSK_PATHWAY	22	0.0087
REACTOME_REGULATORY_RNA_PATHWAYS	21	0.0088
