# Supplementary material for: Sarcoidosis activates diverse transcriptional programs in bronchoalveolar lavage cells
Source: Respir Res. 2016 Jul 26;17:93. doi: 10.1186/s12931-016-0411-y (PMC4962428; doi:10.1186/s12931-016-0411-y)
Supplement: Additional file 6: Table S5. — Subject demographics and BAL differential cell counts (sarcoidosis 2). (PDF 117 kb) [file 12931_2016_411_MOESM6_ESM.pdf]

Additiona file 6: Table S5. Subject demographics and BAL differential cell counts (sarcoidosis 2).

Subject	Gender	Age	Race	Smoking	Mac (%)	Lymph (%)	Pmn (%)	Eos (%)
Sarc 16	F	57	C	Former	69	15	14	2
Sarc 17	F	44	AA	Never	86	11	3	0
Sarc 18	F	27	C	Smoker	95	1	4	0
Sarc 19	F	46	C	Former	93	4	3	0
Sarc 20	M	59	AA	Former	78	10	8	4
Sarc 21	F	24	AA	Smoker	90	9	1	0
Sarc 22	F	37	AA	Former	89	7	4	0
Sarc 23	F	44	AA	Never	79	21	0	0
Sarc 24	M	49	C	Never	95	5	0	0
Sarc 25	F	48	AA	Never	100	0	0	0

Sarc: sarcoidosis patient; AA: African American; C: Caucasian; Mac: macrophage; Lymph: lymphocyte; Pmn: polymorphonuclear cell; Eos: eosinophil.
